# A randomized phase II trial to elucidate the efficacy of capecitabine plus cisplatin (XP) and S-1 plus cisplatin (SP) as a first-line treatment for advanced gastric cancer: XP ascertainment vs. SP randomized PII trial (XParTS II)

**DOI:** 10.1186/1471-2407-12-307

**Published:** 2012-07-23

**Authors:** Akira Tsuburaya, Satoshi Morita, Yasuhiro Kodera, Michiya Kobayashi, Kohei Shitara, Kensei Yamaguchi, Takaki Yoshikawa, Kazuhiro Yoshida, Shigefumi Yoshino, Jun-ichi Sakamoto

**Affiliations:** 1 Department of Gastrointestinal Surgery, Kanagawa Cancer Center, 1-1-2 Nakao, 241-0815, Yokohama, Asahi-ku, Japan; 2 Department of Biostatistics and Epidemiology, Yokohama City University Medical Center, Yokohama, Japan; 3 Department of Surgery II, Nagoya University Graduate School of Medicine, Nagoya, Japan; 4 Department of Surgery, Kochi Medical School, Kochi, Japan; 5 Department of Clinical Oncology, Aichi Cancer Center Hospital, Nagoya, Japan; 6 Department of Gastroenterology, Saitama Cancer Center, Saitama, Japan; 7 Department of Surgical Oncology, Gifu Graduate School of Medicine, Gifu, Japan; 8 Department of Digestive Surgery and Surgical Oncology, Yamaguchi University Graduate School of Medicine, Ube, Japan; 9 Epidemiological and Clinical Research Information Network, Nagoya University Graduate School of Medicine, Aichi, Nagoya, Japan

**Keywords:** Biomarker, Capecitabine, Cisplatin, Clinical trial, Gastric cancer, S-1

## Abstract

**Background:**

On the basis of international clinical trials, capecitabine plus cisplatin (XP) as a first-line treatment of advanced gastric cancer is considered a global standard regimen. However, the usefulness of XP as compared with S-1 plus cisplatin (SP), which is considered standard therapy in Japan, has not yet been assessed.

**Methods/design:**

This is a multicenter randomized phase II trial to elucidate the efficacy of XP as compared with SP for first-line treatment of advanced gastric cancer. Patients with unresectable metastatic or recurrent gastric cancer, 20–74 years of age and human epidermal growth factor 2 (HER2)-negative status, will be assigned in a 1:1 ratio to receive either S-1 40 mg/m^2^ bid for 21 days plus cisplatin 60 mg/m^2^ (day 8) every 5-week cycle or capecitabine 1000 mg/m^2^ bid for 14 days plus cisplatin 80 mg/m^2^ (day 1) every 3-week cycle. Patients will be also asked to the analysis of tumor tissues for translational investigations. The Primary endpoint is progression-free survival and secondary endpoints are overall survival, time to treatment failure, tumor response rate and safety. These comparisons will also be evaluated in terms of biomarkers. Planned sample size is 100 (50 in each arm), which is appropriate for this trial.

**Discussion:**

Fluoropyrimidine plus cisplatin combination is the standard regimen of the first line treatment for advanced gastric cancer. Both S-1 and capecitabine are the prodrug of 5-FU but differ from their process of metabolism. Result of this trial and translational research will provide the important clues to prepare the individualized therapy for advanced gastric cancer in the near future.

**Trial registration:**

ClinicalTrials.gov Identifier NCT01406249

## Background

Gastric cancer is the fourth most common malignancy in the world (988 602 cases in 2008, 7.8% of total) and the second leading cause of cancer death (737 419 deaths, 9.7% of total) [1]. For the treatment of advanced or recurrent gastric cancer (AGC), the most commonly used regimens are combination chemotherapy consisting of a fluoropyrimidine (5-fluorouracil or oral fluoropyrimidine) plus a platinum agent with or without docetaxel or anthracyclines [2-6].

S-1 is an oral anticancer drug composed of the 5-fluorouracil (5-FU) prodrug tegafur and two 5-FU modulators; it has achieved high response rates in patients with gastric cancer in phase II studies [7,8]. In a phase III trial (SPIRITS trial) that compared S-1 alone to S-1 plus cisplatin (SP), SP showed a significantly longer overall survival (OS; 13 months vs. 11 months; HR = 0.77, 95% CI 0.61–0.98, p = 0.04) and longer progression-free survival (PFS; 6.0 months vs. 4.0 months; HR = 0.57, 95% CI 0.44–0.73, p < 0.0001) [4]. Therefore, SP is now considered to be one of the standard first-line regimens for AGC in Japan.

Capecitabine is also an oral fluoropyrimidine, which is metabolized primarily in the liver and converted in tumor tissues to 5-FU by the enzyme thymidine phosphorylase (TP), which is associated in higher concentrations in tumor cells than in normal cells [9]. Kang and colleagues evaluated the non-inferiority of capecitabine plus cisplatin (XP) compared with 5-FU plus cisplatin (FP). The median PFS showed significant non-inferiority (5.6 months vs. 5.0 months; HR = 0.81, 95% CI 0.63–1.04, P < 0.001) [5]. On the basis of these results, XP is now considered one of the standard treatments of AGC [10], and XP was adopted as the reference arm in two recent global studies of molecular targeting agents [11,12]. However, data is scarce with respect to XP treatment in Japanese patients, and also the usefulness of XP as compared with SP has not yet been assessed.

As another issue, these 2 types of oral fluoropyrimidine show some different characteristics in the mechanisms of their antitumor effect. A subset analysis of the FLAGS trial showed that S-1 seemed to be better than 5-FU in the subgroup with diffuse-type gastric cancer [6]. This result was consistent with the results of a subset analysis of the JCOG9912 trial, which showed that S-1 was better than 5-FU in patients with diffuse-type gastric cancer or with gastric cancer associated with high dihydropyrimidine dehydrogenase (DPD), with diffuse-type tumors associated more commonly than intestinal type with high DPD [13]. This result was expected, since S-1 consists of tegafur, otastat potassium, and gimestat which is a potent competitive inhibitor of DPD. Capecitabine is transformed to 5-FU in several steps, to be finally converted by TP as above [9]. A phase II trial in Japan showed that response rate (RR) was significantly higher (Fisher’s exact test, *p* = 0.028) in patients with TP-positive and DPD-negative tumors (60%, 6/10) than in the remaining patients (13%, 2/15) [14]. In contrast, high expression of TP is reported to be negatively associated with efficacy of 5-FU or S-1 in gastric cancer [15,16].

On the basis of the above reports, histological type (diffuse or intestinal) and biomarkers (TP, DPD, and others) may be candidates to select whether S-1 or capecitabine be used for each patient, although validation with a randomized study is necessary. We planned the current clinical trial to elucidate the efficacy of XP and SP for the first-line treatment of AGC. This comparison will be also evaluated in terms of several biomarkers.

## Method/design

### Study objective

This randomized phase II trial is planned to elucidate the efficacy of SP and XP and also to explore predictive or prognostic biomarkers with additional research. This trial protocol has been approved by the Institutional Review Board (IRB) of each participating institution and the Kanagawa Cancer Center.

### Study endpoints

Primary endpoint is PFS and secondary endpoints are OS, RR, time to treatment failure (TTF), and incidence of adverse events (safety).

### Eligibility criteria

#### Inclusion criteria

(i) Histologically confirmed gastric adenocarcinoma with unresectable metastatic or recurrent disease

(ii) Lesions confirmed by imaging no more than 28 days before registration (not required for measurable lesions as defined in RECIST version 1.1)

(iii) No previous chemotherapy or radiotherapy. However, prior adjuvant chemotherapy is allowed if more than 6 months has passed since the end of adjuvant chemotherapy

(iv) Eastern Cooperative Oncology Group (ECOG) Performance Status of 0, 1, or 2

(v) Life expectancy of at least 3 months after registration

(vi) Written informed consent

(vii) Between the ages of 20 and 74 years at the time informed consent is obtained

(viii) Adequate major organ function including:

(a) Neutrophil count: ≥1500/mm^3^

(b) Platelet count: ≥10.0 × 10^4^/mm^3^

(c) Hemoglobin: ≥9.0 g/dL

(d) AST, ALT: ≤2.5 × upper limit of normal (ULN) in each institution (≤5 times in cases of metastases to liver)

(e) ALP: ≤2.5 × ULN in each institution (≤5 times in cases of metastases to liver, and ≤10 times in cases of metastases to bone)

(f) Total bilirubin: ≤1.5 × ULN in each institution

(g) Creatinine clearance: ≥60 mL/min (as estimated by Cockcroft-Gault equation)

#### Exclusion criteria

(i) HER2- positive status

(ii) Previous history of fluoropyrimidine therapy within 6 months prior to registration

(iii) Previous treatment with platinum agents within 12 months prior to registration

(iv) Previous treatment with cisplatin more than total dose of 120 mg/m^2^

(v) Previous history of serious hypersensitivity to fluoropyrimidines or platinum agents

(vi) Previous history of adverse reactions suggestive of dihydropyrimidine dehydrogenase (DPD) deficiency

(vii) More than 1 cancer at the same time or more than 1 cancer at different times separated by a 5-year disease-free interval. However, multiple active cancers do not include carcinoma *in situ* or skin cancer which is determined to have been cured as a result of treatment.

(viii) Obvious infection or inflammation (pyrexia ≥38.0°C)

(ix) Active hepatitis

(x) Heart disease that is serious or requires hospitalization, or history of such disease within the past year

(xi) Having a complication that is serious or requires hospitalization (intestinal paralysis, intestinal obstruction, interstitial pneumonia or pulmonary fibrosis, poorly controlled diabetes mellitus, renal failure, liver disorders, or hepatic cirrhosis)

(xii) Being treated or in need of treatment with flucytosine, phenytoin, or warfarin potassium

(xiii) Chronic diarrhea (watery stools or ≥4 times/day)

(xiv) Active gastrointestinal bleeding

(xv) Body cavity fluids requiring drainage or other treatment

(xvi) Clinical suspicion or previous history of metastasis to brain or meninges

(xvii) Women who are pregnant, breastfeeding, or potentially (hoping to become) pregnant

(xviii) Unwillingness to practice contraception

(xix) Poor oral intake

(xx) Psychiatric disorders which are being, or may need to be, treated with psychotropics

(xxi) Otherwise determined by investigators or site principal investigators to be unsuitable for participation in study

### Registration

Physicians or coordinators will send a Case Registration Form to the data center (Epidemiological and Clinical Research Information Network, ECRIN) with all the required items filled out. Enrollment has started from July 2011.

### Startification

Eligible patients will be randomized to either Arm-A (SP treatment) or Arm-B (XP treatment) by dynamic allocation via a centralized randomization method using 5 stratification factors as balancing variables:

(i) baseline ECOG Performance Status (0–1/2)

(ii) measurable lesion (yes/no)

(iii) prior adjuvant chemotherapy (yes/no)

(iv) histopathological classification (intestinal/diffuse)

(v) institution.

### Statistical analysis

PFS has been set as the primary endpoint and is defined as the time from date of registration until the date that progression is determined or the date of death for any reason, whichever is sooner. “Progression” will be evaluated on the basis of Response Evaluation Criteria In Solid Tumors (RECIST) version 1.1 [17]. More information about the definition of PFS and Progression are pre-specified (Table 1).

**Table 1:** Definition of PFS and progression

**Definition of PFS and progression are predefined as below**	
1.)	PFS will be determined as the time from the date of registration until the date that progression is determined or the date of death for any reason, whichever is sooner.
2.)	“Progression (PD)” means both PD confirmed by routine diagnostic imaging in each course and PD confirmed by as-needed diagnostic imaging in the case that there is clinical suspicion of PD. In the latter case, it is preferable that there is at least objective evidence.
3.)	When progression is determined based on diagnostic imaging, the date of progression will be the date on which imaging is assessed. When clinical progression is first determined independently of diagnostic imaging, and then later objectively determined on the basis of diagnostic imaging, the date of progression will be back-dated to the date of determination of clinical progression. If no objective evidence is obtained, it will be treated as a censoring event in the formal analysis, and sensitivity analysis will be also conducted as if this were PD.
4.).	When considering tumor regrowth and determining PD according to RECIST, it is considered a PD as PFS event regardless of tumor diameter. But even if it is decided as PD according to RECIST, investigators can continue the protocol treatment if they consider continued treatment to be beneficial to the patient.
5.)	If treatment discontinuation is needed due to symptomatic deterioration without any objective evidence at that time, it is reported as “symptomatic deterioration”. Investigators should endeavor to obtain objective evidence of the progression even after discontinuation of treatment. In this case, the event shall be judged to be clinical PD and handled as mentioned in 2) above. When progression is determined on the basis of diagnostic imaging, the date of progression will be back-dated to the date of diagnosis of symptomatic deterioration.
6.)	Survivors for whom progression has not been determined will be censored based on the last date on which the absence of progression was clinically confirmed (the last day that PFS was confirmed).
7.)	Cases of discontinuation of protocol treatment because of toxicity or patient refusal, even if another therapy is added as a post-treatment, will be censored at the date of discontinuation or the date that post-treatment was started.
8.)	In cases where progression is diagnosed on the basis of imaging, the event will be determined based not on evaluation dates where the result is “suspected” on imaging but on a subsequent evaluation date where progression is “confirmed” on imaging.
9.)	Secondary cancer (multiple cancers in metachronous) will not be regarded as either an event or censored.

The primary objective of this trial is to evaluate the PFS of SP and XP as the first-line treatment for advanced gastric cancer. The 24-week progression-free rate (PFR) will be estimated for each group, calculating point estimates and 2-sided 90% confidence intervals. The 2-sided 90% confidence interval of the difference between the 2 groups will be also estimated. Exploratory analysis will be done to test the null hypothesis that PFS is equal in both groups. Cumulative PFS curves will be constructed as time-to-event plots by the Kaplan-Meier method.

With respect to secondary endpoints, efficacy endpoints OS and TTF will be evaluated according to the method of analysis of the primary endpoint. Overall response rate (RR) is defined as the proportion of patients with complete response (CR) or partial response (PR) by RECIST out of the patients with measurable lesions, and the chi-square test will be used to compare the 2 groups. The 2-sided 95% confidence interval of the difference between the 2 groups will also be estimated. For the analysis of safety, Fisher’s exact test will be used if necessary, and the exact confidence intervals for the binomial distribution will be estimated.

### Sample-size calculation

Assuming a threshold 24-week PFR of 40% and an expected 24-week PFR of 55% (clinically promising), and a 1.5-year registration period and a 1.5-year follow-up period, 49 patients are required in each group to ensure a 1-sided alpha of 5% and statistical power of 90%. Assuming that the 24-week PFR of the biomarker-positive (any FU-related enzyme or expression of intestinal type) population in the SP arm is 45%, and the risk reduction rate in the XP arm is 40%, 46 patients in total are needed to ensure a 2-sided alpha of 10% and statistical power of 70%. Under the hypothesis that the targeted biomarker-positive population is 50%, 92 patients in total are required. Considering the likelihood of some ineligible cases in the whole setting outlined above, the total sample size is set to 100. A following Phase III study will be designed for both randomized comparison and biomarker-oriented comparison of XP and SP (4 groups).

### Treatment program

Patients who allocated SP will be treated with S-1 and cisplatin every 5-week cycle. S-1 will be administered orally at a dose of 40 mg/m^2^ twice-daily (equivalent to a total daily dose of 80 mg/m^2^) for 3 weeks (day 1 to 21). Cisplatin 60 mg/m^2^ on day 8 of each cycle will be given by intravenous infusion over 2 hours. On the other hand, patients who allocated XP will be treated with capecitabine and cisplatin every 3-week cycle. Capecitabine will be administered orally at a dose of 1000 mg/m^2^ twice-daily (equivalent to a total daily dose of 2000 mg/m^2^) for 2 weeks (day 1 to 14). Cisplatin 80 mg/m^2^ on day 1 of each cycle will be given by intravenous infusion over 2 hours.

Treatment continuation is intended until disease progression or unacceptable toxicity. If treatment continuation with cisplatin is determined to be unfeasible before any progression is confirmed, continuously monotherapy of S-1 or capecitabine will be continued until PD.

### Follow-up

During treatment under this protocol, patients will have a physical check-up and a blood examination before every drug administration. PFS and RR will be monitored by using abdominal CT or MRI every 6 weeks and by measuring levels of tumor markers CEA and CA19-9.

### Translational research project

Translational research will be conducted to elucidate the clinical utility of the following biomarkers. These biomarkers will be analyzed Immunohistochemistry (IHC) and mRNA expression by using tissue specimen. Tumor tissue samples from primary lesions and/or biopsy material will be collected and centralized assessment.

(i) Immunohistochemistry (IHC): Expression of TP, DPD, ERCC1, Ki67, LGALS4, and CDH17

(ii) mRNA: Expression of TP, DPD, thymidylate synthase (TS), orotate phosphoribosyltransferase (OPRT), and excision repair cross-complementation group1 (ERCC1)

## Discussion

Recently, molecular target drugs has resulted in the opportunity to provide individualized treatment in the field of AGC. Especially in patients with HER2-positive AGC (defined as assessed by IHC 3+ on a scale of 0 to 3+, and/or fluorescence in-situ hybridization; FISH, *HER2*:CEP17 ratio ≥2.0), ToGA study showed that adding trastuzumab was significantly improved overall survival comparing with standard chemotherapy consists of cytotoxic drugs [11]. This study excludes HER2-positive gastric cancer since these patients should be recommended trastuzumab containing regimen. The individualized treatment for cytotoxic agents also needs to be developed to have more effect and less toxicity.

This is the first study to compare two standard regimens for AGC. Additionally, the translational research is performed to explore the biomarker for chemo-sensitivity and make the individualized treatment possible. When the difference of treatment is found in efficacy or safety from this analysis, we will conduct a phase III trial to examine the possibility of individualized treatment. We believe the result of this study will play the important role to prepare the individualized therapy for advanced gastric cancer in the near future.

## Competing interests

All authors declare that they have no competing interest.

## Authors’ contributions

AT drafted the manuscript and wrote the original protocol for the study. All authors participated in the design of the study. SM performed the statistical analysis. All authors read and approved the final manuscript.

## Pre-publication history

The pre-publication history for this paper can be accessed here:

http://www.biomedcentral.com/1471-2407/12/307/prepub
